# Specific Inhibition of Complement Activation Significantly Ameliorates Autoimmune Blistering Disease in Mice

**DOI:** 10.3389/fimmu.2018.00535

**Published:** 2018-03-16

**Authors:** Sidonia Mihai, Misa Hirose, Yi Wang, Joshua M. Thurman, V. Michael Holers, B. Paul Morgan, Jörg Köhl, Detlef Zillikens, Ralf J. Ludwig, Falk Nimmerjahn

**Affiliations:** ^1^Lübeck Institute of Experimental Dermatology and Department of Dermatology, University of Lübeck, Lübeck, Germany; ^2^Institute of Genetics, Department of Biology, University of Erlangen-Nuremberg, Erlangen, Germany; ^3^Alexion Pharmaceuticals, Cheshire, CT, United States; ^4^Departments of Medicine and Immunology, University of Colorado Health Sciences Center, Denver, CO, United States; ^5^Systems Immunity Research Institute, School of Medicine, Cardiff University, Cardiff, United Kingdom; ^6^Institute for Systemic Inflammation Research, University of Lübeck, Lübeck, Germany; ^7^Division of Immunobiology, Cincinnati Children’s Hospital Medical Center and University of Cincinnati, College of Medicine, Cincinnati, OH, United States

**Keywords:** autoantibodies, complement, complement inhibition, immunotherapy, autoimmune diseases

## Abstract

Epidermolysis bullosa acquisita (EBA) is an antibody-mediated blistering skin disease associated with tissue-bound and circulating autoantibodies to type VII collagen (COL7). Transfer of antibodies against COL7 into mice results in a subepidermal blistering phenotype, strictly depending on the complement component C5. Further, activation predominantly by the alternative pathway is required to induce experimental EBA, as blistering was delayed and significantly ameliorated only in factor B^−/−^ mice. However, C5 deficiency not only blocked the activation of terminal complement components and assembly of the membrane attack complex (MAC) but also eliminated the formation of C5a. Therefore, in the present study, we first aimed to elucidate which molecules downstream of C5 are relevant for blister formation in this EBA model and could be subsequently pharmaceutically targeted. For this purpose, we injected mice deficient in C5a receptor 1 (C5aR1) or C6 with antibodies to murine COL7. Importantly, *C5ar1*^−/−^ mice were significantly protected from experimental EBA, demonstrating that C5a–C5aR1 interactions are critical intermediates linking pathogenic antibodies to tissue damage in this experimental model of EBA. By contrast, *C6*^−/−^ mice developed widespread blistering disease, suggesting that MAC is dispensable for blister formation in this model. In further experiments, we tested the therapeutic potential of inhibitors of complement components which were identified to play a key role in this experimental model. Complement components C5, factor B (fB), and C5aR1 were specifically targeted using complement inhibitors both prophylactically and in mice that had already developed disease. All complement inhibitors led to a significant improvement of the blistering phenotype when injected shortly before anti-COL7 antibodies. To simulate a therapeutic intervention, anti-fB treatment was first administered in full-blown EBA (day 5) and induced significant amelioration only in the final phase of disease evolution, suggesting that early intervention in disease development may be necessary to achieve higher efficacy. Anti-C5 treatment in incipient EBA (day 2) significantly ameliorated disease during the whole experiment. This finding is therapeutically relevant, since the humanized anti-C5 antibody eculizumab is already successfully used in patients. In conclusion, in this study, we have identified promising candidate molecules for complement-directed therapeutic intervention in EBA and similar autoantibody-mediated diseases.

## Introduction

Pemphigoid diseases (PD), such as bullous pemphigoid (BP) and epidermolysis bullosa acquisita (EBA) are prototypical, antibody-mediated autoimmune diseases of the skin. They are clinically characterized by (muco)-cutaneous inflammation and blistering and caused by autoantibodies against structural proteins of the skin (1–3). Tissue-bound and circulating antibodies in EBA are directed against the noncollagenous domain 1 of type VII collagen (COL7). The pathogenic relevance of antibodies to COL7 was demonstrated through multiple lines of experimental evidence: (1) EBA autoantibodies recruit and activate leukocytes *ex vivo* and induce dermal–epidermal separation in cryosections of human skin (4, 5). (2) Passive transfer of antibodies to COL7 (6, 7) and immunization with recombinant COL7 (8) in mice results in a blistering phenotype closely resembling human EBA.

In recent years, use of animal models has significantly contributed to the understanding of PD pathogenesis. Three major checkpoints lead to PD: first, loss of tolerance to PD antigens leads to the CD4- and neutrophil-dependent generation of antigen-specific plasma cells. Second, autoantibodies are released into the circulation, where the half-life of IgG autoantibodies is controlled by the neonatal Fc receptor. Third, in the effector phase, autoantibodies bind to their target antigens located in the skin, which leads to the formation of a pro-inflammatory milieu allowing an ICAM-1- and CD18-dependent extravasation of myeloid effector cells into the skin. Within the skin, myeloid cells bind to the immune complexes *via* specific Fc gamma receptors (FcγRs), become activated, and release reactive oxygen species and proteases, which ultimately facilitate inflammation and blistering (9, 10).

Intriguingly, activation of the complement system has emerged as a key requirement to mediate inflammation and blistering in PD. Specifically, in antibody transfer models of BP and EBA, mice lacking the complement component C5 failed to develop clinically significant experimental PD (6, 11, 12). Upstream of C5, complement activation by both classical and alternative pathway is required to induce clinical manifestations. Interestingly, induction of experimental BP mainly depended on classical complement activation, whereas the alternative pathway predominantly drives inflammation in experimental EBA. Regarding the lectin-pathway, MBL-null mice developed a blistering phenotype similar to the wild-type control animals in experimental EBA, while no data has been published in this context relating to BP (13, 14).

C5 is cleaved by the C5 convertase into C5a and C5b fragments. “When C5b associates with C6 and C7, the complex inserts into cell membranes and interacts with C8, inducing the binding of several units of C9 to form a lytic pore, the terminal membrane complex (C5b-9, also known as the membrane attack complex, MAC). Many pathogens are protected from MAC-mediated lysis through their cell wall architecture or by employing evading strategies that interfere with MAC assembly. However, even sublytic amounts of MAC or partial complexes such as C5b-8 drive nonlethal signaling events. Pro-inflammatory signaling and phagocytosis are essential for complement-mediated defense. During activation and amplification, C5a is constantly released and triggers strong pro-inflammatory signaling mainly through its corresponding G-protein-coupled receptor C5a receptor 1 (C5aR1, CD88), guiding neutrophils, monocytes, and macrophages toward sites of complement activation” [reviewed in Ref. (15)]. C5a also binds to the more recently discovered seven transmembrane receptor C5aR2 (C5L2, GPR77), which is uncoupled from G proteins (16). However, the exact biologic function of this C5aR is not yet fully determined. Depending on the experimental setting it exerts either decoy, regulatory or even pro-inflammatory functions (15, 17). Its role on EBA development has not been explored. We recently showed that C5aR1-deficient mice are almost completely protected from inflammation and blistering in antibody transfer-induced EBA (18). It was, however, so far unclear whether the membrane attack complex (MAC) also contributes to skin blistering in EBA, and if pharmacological targeting of complement can ameliorate the EBA effector phase. Current evidence suggests that generation of C5a and the formation of MAC are both essential for triggering pro-inflammatory responses in disease models like collagen-antibody-induced arthritis (19) and renal ischemia/reperfusion injury (20, 21). This lack of clarity may be particularly relevant to the use of the C5 antibody eculizumab in PD (22). To address these knowledge gaps, we here systematically evaluated the contribution of complement components downstream of C5 and evaluated the therapeutic potential of targeting the implicated complement proteins in both preventive and therapeutic settings.

## Materials and Methods

### Mice

*C5ar1*^−/−^ mice (*n* = 10) backcrossed to BALB/c mice were acquired from The Jackson Laboratory. *C6*^−/−^ mice (*n* = 6) backcrossed to C57BL/6J mice were kindly provided by Prof. B. Paul Morgan, Cardiff University, UK. The *C6*^−/−^ C57BL/6J mice were derived from a *C6*^−/−^ C3H/He mouse strain lacking functional C6 (23). Age-matched female BALB/c and C57BL/6J mice were purchased from Charles River (Sulzfeld, Germany). Female mice, at 6–8 weeks of age, on the BALB/c background (*n* = 4/group), obtained from Charles River (Sulzfeld, Germany) were used for all complement-blocking experiments. Anesthesia in mice was induced by inhalation of isoflurane or intraperitoneal administration of a mixture of ketamine (100 µg/g) and xylazine (15 µg/g). The experiments were approved by the local authorities of the Animal Care and Use Committee (6/v/08) and performed by certified personnel.

### Affinity-Purification of Antibodies

Total IgG from rabbits immunized with murine COL7c was purified by affinity chromatography using protein G affinity as previously reported and reviewed (6, 24). Before use in preclinical models, reactivity of IgG fractions was analyzed by immunofluorescence (IF) microscopy on murine skin.

### EBA Induction

Passive transfer studies followed published protocols with minor modifications (6). Briefly, mice received six injections of 10 mg rabbit IgG every second day, over a period of 12 days. Skin affected by blisters and/or erosions was quantified as a percentage of the total mouse body surface area, as extensively described in Ref. (24). The evaluation of the affected body area was not performed by blinded observers. At the end of the experiment (day 12), mice were sacrificed and skin biopsies were taken for examination by histopathology and IF microscopy (to detect tissue-bound IgG and complement C3 deposits), as described (6). Deposition of murine C5 was detected by incubation of cryosections of perilesional skin with a monoclonal antibody specific to murine C5 (BB5.1) (25) and, finally, with a FITC-labeled antibody specific to mouse IgG (DAKO, Glostrup, Denmark), as described (6).

### Complement Inhibition

The alternative pathway of complement activation was inhibited by a monoclonal antibody to murine factor B (fB) (clone 1379) (26). For prophylactic application, mice were pre-injected intraperitoneally (ip) daily with 2 mg anti-fB or control mouse polyclonal IgG, and, subsequently, with 10 mg of purified anti-COL7 IgG, subcutaneously, every second day, for 12 days. For the therapeutic approach, mice were treated with six injections of 10 mg anti-COL7 IgG. Starting with day 5, when cutaneous lesions were clearly visible in both groups, mice were additionally injected ip daily with anti-fB or control mouse IgG. For C5-inhibition, a monoclonal antibody to murine C5 (clone BB5.1) was used (25). In a first approach, mice were pre-injected ip twice a week with 40 mg/kg of anti-C5 mAb or an isotype control (HFN7.1), and, subsequently, with 10 mg anti-COL7 IgG, every second day, for 12 days. For the therapeutic approach, mice were injected with six doses of pathogenic rabbit IgG. 24 h after the first anti-COL7 IgG injection and then twice a week (on days 4, 7, and 10) mice were additionally injected with anti-murine C5 or an isotype control. C5aR1 was pharmacologically targeted using a C5aR1/2 antagonist A8Δ71–73 (C5aRA) (27). Mice were injected subcutaneously with 10 mg anti-COL7 IgG, every second day, for 12 days. One group of mice was pretreated daily with 10^−5^M C5aRA, intravenously for the first 5 days and ip for the rest of the experiment.

### Statistical Analysis

Differences in disease severity were calculated using the Mann–Whitney *U*-test. Means are presented ±SEM; *p* < 0.05 was considered statistically significant.

## Results

### C5aR1-Deficient Mice Are Protected From Induction of Experimental EBA

**Figure 1:**
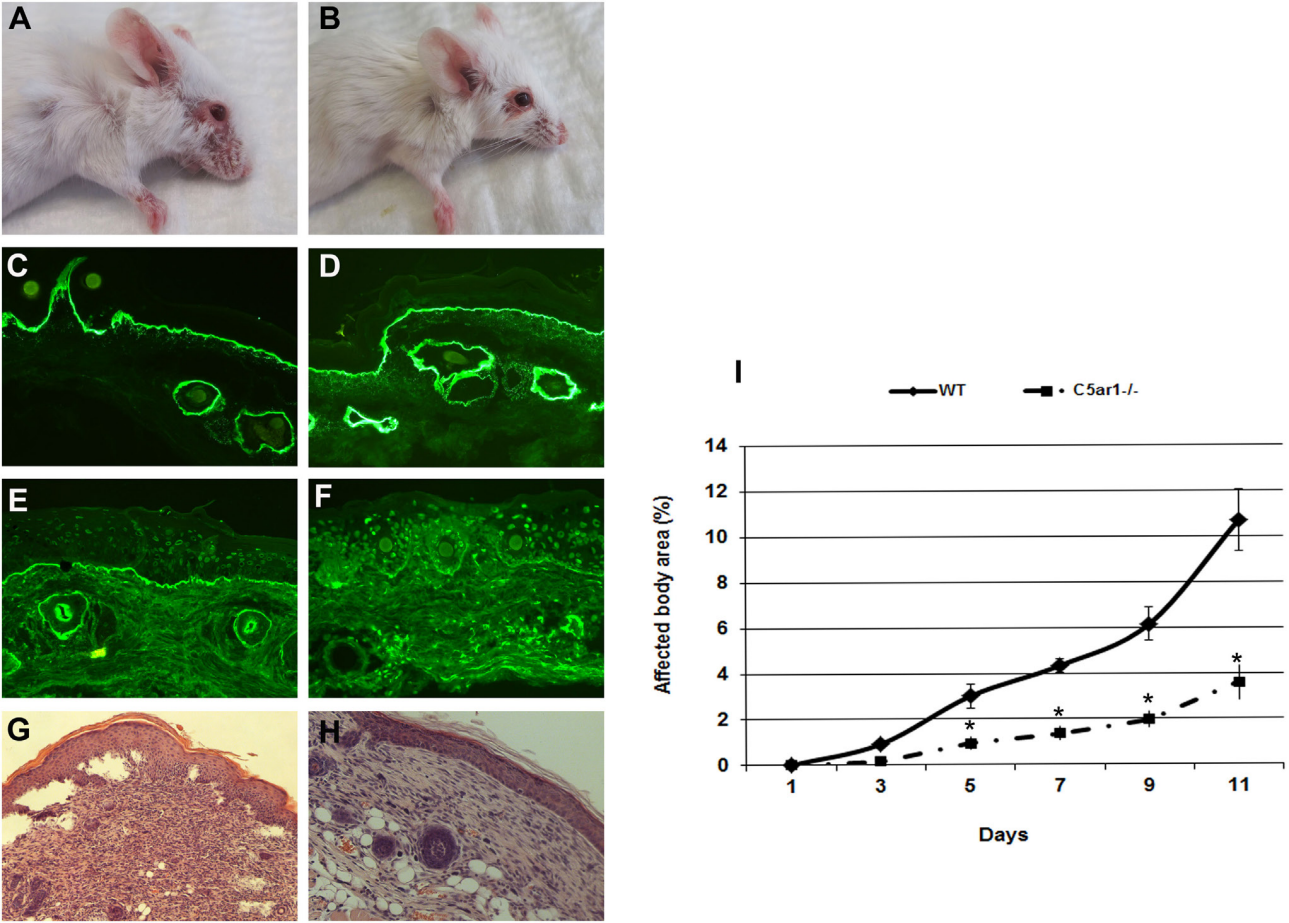
C5a receptor 1 (C5aR1)-deficient mice are protected from blister induction in experimental epidermolysis bullosa acquisita. Representative day 12 clinical pictures showing extensive skin lesions, including blisters, erosions, partly covered by crusts on the ear and front leg, and alopecia of snout and around the eyes in a control mouse **(A)**, in contrast to a C5aR1-deficient mouse receiving the same dose of anti-type VII collagen (COL7) IgG **(B)**. Immunofluorescence analysis revealed linear deposition of rabbit IgG **(C,D)** at the dermal–epidermal junction in all mice injected with anti-COL7. Deposition of murine C5 was strong in the skin of the control mouse **(E)** and weak or absent in the skin of the C5aR1-deficient mouse **(F)**. Histological analysis of lesional murine skin revealed subepidermal cleavage and a neutrophil-rich inflammatory infiltrate in the skin of the control mouse **(G)** and to a lesser extent, in the **(H)** skin of the C5aR1-deficient mouse defiecient mouse. Affected body area of C5aR1-deficient mice vs control mice is represented as mean ± SEM. *p < 0.05 represents significant difference of disease activity between the two groups **(I)**. These data were obtained from two independent experiments (*n* = 5/group/experiment).

The anaphylatoxin C5a is a powerful chemoattractant for neutrophils, mast cells, and monocytes, and it exerts many of its biologic functions exclusively by activation of C5aR1 (17). Leukocyte recruitment to the dermal–epidermal junction by antibodies and release of inflammatory mediators are prerequisites for blister induction in this animal model (28). To study the C5a–C5aR1 interactions, *C5ar1*^−/−^ and control mice were injected with rabbit antibodies to murine COL7 to induce experimental EBA. C5aR1-deficient mice were significantly protected from blister induction (Figure 1), confirming that C5a–C5aR1 interactions are critical intermediates linking pathogenic antibodies to tissue damage in this experimental model of EBA.

### C6-Deficient Mice Are Susceptible to Induction of Experimental EBA

**Figure 2:**
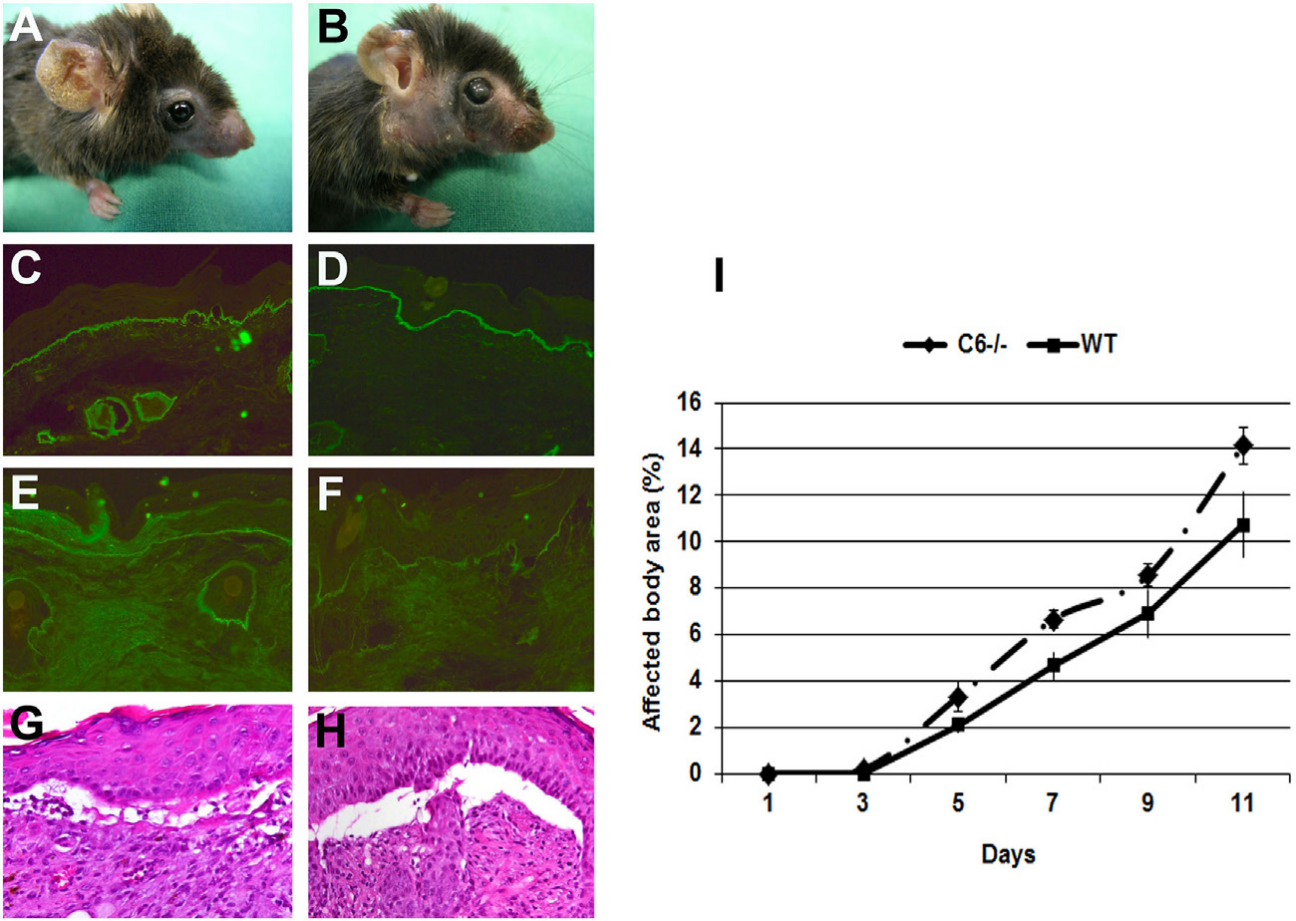
C6-deficient mice are susceptible to inflammation and blistering in experimental epidermolysis bullosa acquisita. Skin lesions, including blisters and erosions covered by crusts on the ear and front leg, and alopecia of the snout and around eyes developed in both **(A)** C6-sufficient and **(B)** C6-deficient mice (day 12). Immunofluorescence analysis of perilesional skin revealed deposition of rabbit IgG **(C,D)** and murine C3 **(E,F)** at the dermal–epidermal junction of both C6-sufficient and C6-deficient mice. Histological analysis of lesional skin revealed extensive dermal–epidermal separation in both C6-sufficient **(G)** and C6-deficient mice **(H)**. Affected body area of C6-deficient mice vs control mice is represented as mean ± SEM **(I)**. These data were obtained from a single experiment (*n* = 6/group).

In the skin of EBA patients, deposition of different complement components, including MAC, are found with an incidence ranging from 40 to 100%. In addition, direct IF microscopy of perilesional skin showed linear deposits of MAC at the dermal–epidermal junction of mice injected with antibodies to murine COL7 (6). C6 is the first among terminal complement components that associates with C5b to initiate the assembly of MAC. To study the implication of complement in tissue destruction *via* C5b-9, mice deficient in C6 and wild-type mice were injected with rabbit antibodies to murine COL7. Both C6-deficient and control mice developed widespread blistering disease (Figure 2), to a similar extent, indicating that MAC is dispensable for blister formation in this model.

### Therapeutic Complement Inhibition in Experimental EBA

In further experiments, we aimed to evaluate the efficiency of novel therapeutic approaches using complement inhibitors. To test their prophylactic potential, mice were pretreated with complement inhibitors and, subsequently, injected with rabbit antibodies to murine COL7. To test for a potential therapeutic relevance of complement inhibition, mice were injected with complement inhibitors in incipient disease.

### Mice Pretreated With a Monoclonal Antibody to Murine Factor B Develop Less Severe Blistering

**Figure 3:**
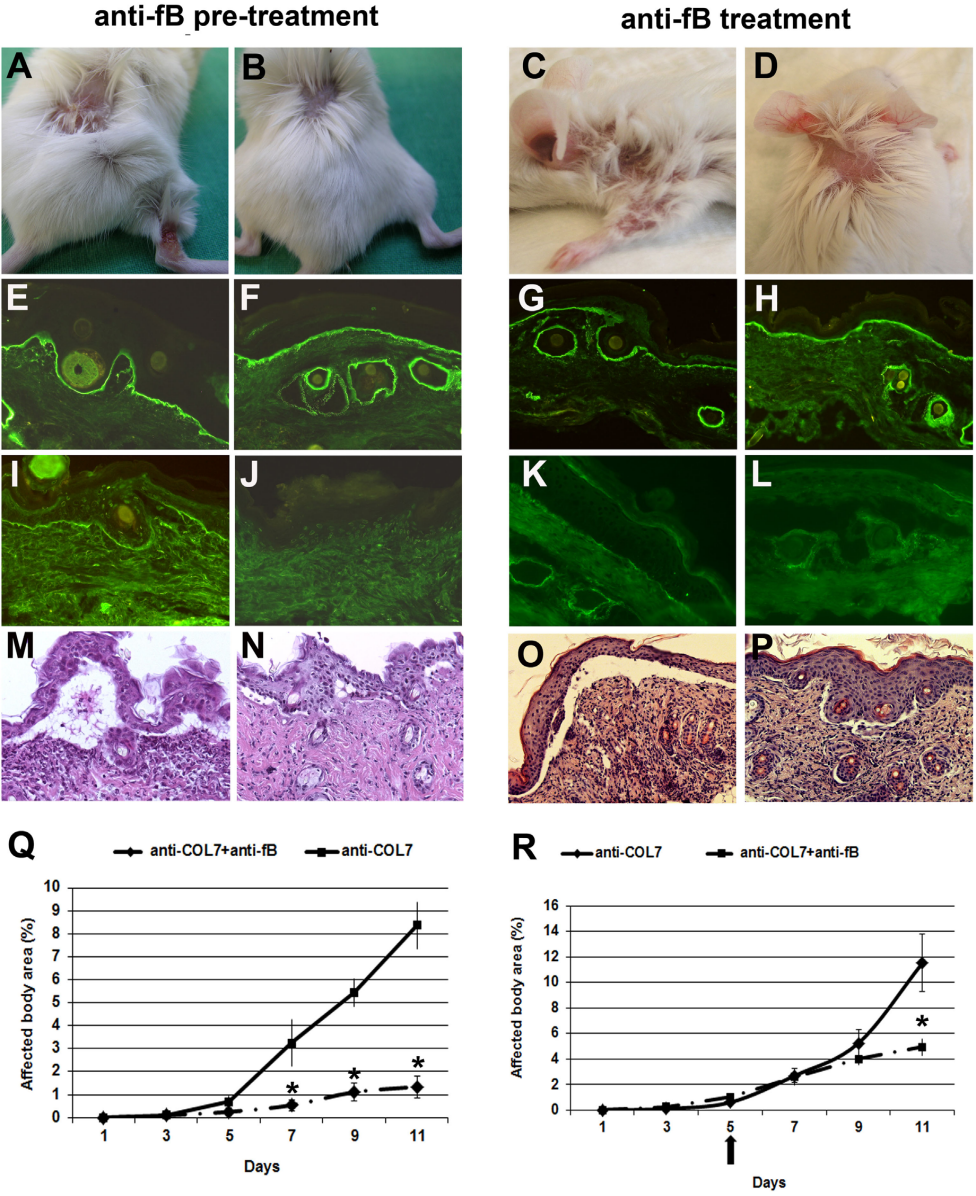
Mice pretreated with a monoclonal Ab to murine factor B (fB) develop a significantly less severe blistering phenotype. BALB/c mice were pretreated with anti-fB (left), followed by injection of anti-type VII collagen (COL7) IgG, and evaluated for skin lesions. To test for a potential therapeutic relevance, mice were injected with anti-fB in incipient disease (right). Erosions covered by crusts on the back and leg of a BALB/c mouse injected with rabbit IgG against murine COL7 **(A,C)**. Less intensive lesions (alopecia) in a **(B,D)** BALB/c mouse challenged with the same dose of pathogenic IgG and a mAb to murine fB. Direct immunofluorescenc analysis of mouse skin revealed deposition of rabbit IgG in mice injected with pathogenic rabbit IgG and **(E,G)** control murine IgG or **(F,H)** anti-murine fB. Deposits of mouse C5 along the dermal–epidermal junction were strong in the skin of the **(I,K)** BALB/c mouse injected with pathogenic rabbit IgG and control murine IgG and absent in the skin of the **(J,L)** BALB/c mouse treated with antibodies to COL7and anti-murine fB. Histological analysis revealed extensive dermal–epidermal separation and inflammatory infiltrates, consisting mainly of neutrophils, in the skin of the control mice **(M,O)** and, to a lesser extent, in the skin of the treated mice **(N,P)**. Affected body area of anti-fB treated vs control mice is represented as mean ± SEM. *p < 0.05 represents significant difference of disease activity between the two groups **(Q,R)**. The arrow indicates the beginning of anti-fB treatment. These data were obtained from a single experiment (*n* = 4/group).

We have previously demonstrated that fB-deficient mice are significantly protected from blistering when injected with pathogenic antibodies to murine COL7 (14). To pharmacologically inhibit the alternative pathway of complement activation in this model, we used a blocking mAb to murine fB (clone 1379). In previous studies, this antibody prevented antiphospholipid antibody-induced fetal loss in a murine model dependent upon activation of the alternative complement pathway (26). In a first set of experiments, BALB/c mice were pre-injected with anti-fB or control antibody, and, subsequently, with anti-COL7 IgG. Mice pretreated with anti-fB, in contrast to control mice, developed significantly less severe blistering throughout the whole experiment (Figure 3).

As the prophylactic application of anti-fB impaired induction of blistering, we next tested the potential therapeutic relevance of this antibody. To address this, experimental EBA was induced in BALB/c mice by repetitive applications of anti-COL7 IgG. On day 5 of the experiment, when cutaneous lesions were clearly visible, one group of mice was additionally injected with anti-fB, while the other group was injected with similar amounts of control mouse IgG. Anti-fB treatment in incipient EBA-induced significant amelioration of disease progression (Figure 3). This identifies fB as an additional target of therapy in inflammatory PD.

### Anti-C5 Significantly Ameliorates Blister Formation in Mice

**Figure 4:**
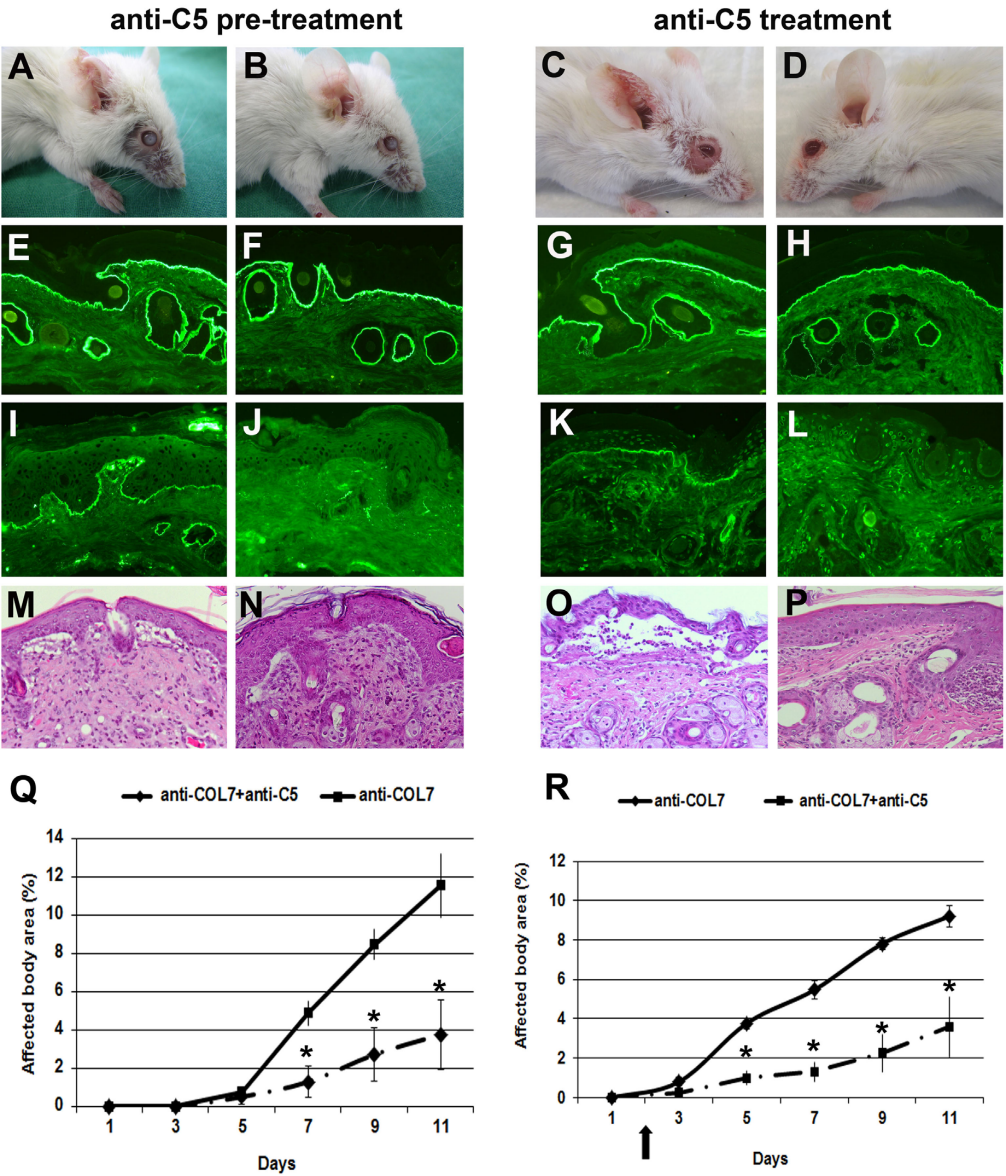
Anti-C5 treatment significantly ameliorates blistering in epidermolysis bullosa acquisita. BALB/c mice were pretreated with anti-C5 (left), followed by injection of anti-type VII collagen (COL7), and evaluated for skin lesions. Alternatively, to test for a potential therapeutic relevance, mice were injected with anti-C5 in incipient disease (right). Injection of rabbit anti-COL7 IgG resulted in extensive skin lesions, including blisters, erosions, partly covered by crusts on the ear and front leg, and alopecia around the eyes in a BALB/c mouse treated with the mock antibody **(A,C)**. A BALB/c mouse receiving the same dose of pathogenic IgG and the anti-murine C5 mAb **(B,D)** showed less extensive lesions. Direct immunofluorescenc analysis revealed linear deposition of rabbit IgG **(E, F, G, H)** at the dermal–epidermal junction in all mice injected with antibodies to COL7. Deposition of murine C5 was strong in the skin of the control mouse **(I,K)** and weak or absent in the skin of the mouse treated with anti-murine C5 antibody **(J,L)**. Histological analysis of lesional murine skin revealed subepidermal cleavage and a neutrophil-rich inflammatory infiltrate in skin of the **(M,O)** BALB/c mouse injected with the mock antibody, and to a lesser extent, in skin of the **(N,P)** BALB/c mouse receiving antibodies to COL7and the blocking anti-C5 antibody. Affected body area of anti-murine C5 treated vs control mice is represented as mean ± SEM. *p < 0.05 represents significant difference of disease activity between the two groups **(Q, R)**. The arrow indicates the beginning of anti-C5 treatment. These data were obtained from a single experiment (*n* = 4/group).

We previously showed that C5-deficient mice are (almost completely) resistant to blister induction by transfer of anti-COL7 IgG (6, 12). To test the prophylactic and therapeutic effects of C5-inhibition, we used a blocking mAb to murine C5 (clone BB5.1) (29). In a first approach, BALB/c mice were pre-injected with anti-C5 or an isotype control (HFN7.1), and, subsequently, with anti-COL7 IgG. In line with the experiments using C5-deficient mice (6), mice treated with anti-C5 developed significantly less blistering disease, during the whole observation period (Figure 4).

In a next set of experiments, we evaluated the potential therapeutic relevance of C5-inhibition. Based on the results obtained with anti-fB, we here initiated treatment at day 2 to evaluate if early treatment may have a more pronounced effect on inflammation and blistering in experimental EBA. Anti-C5 treatment in incipient EBA successfully and significantly ameliorated disease during the entire observation period (Figure 4).

### C5aR1 Inhibition Leads to a Significant Improvement of the Blistering Phenotype at the End of the Observation Period

**Figure 5:**
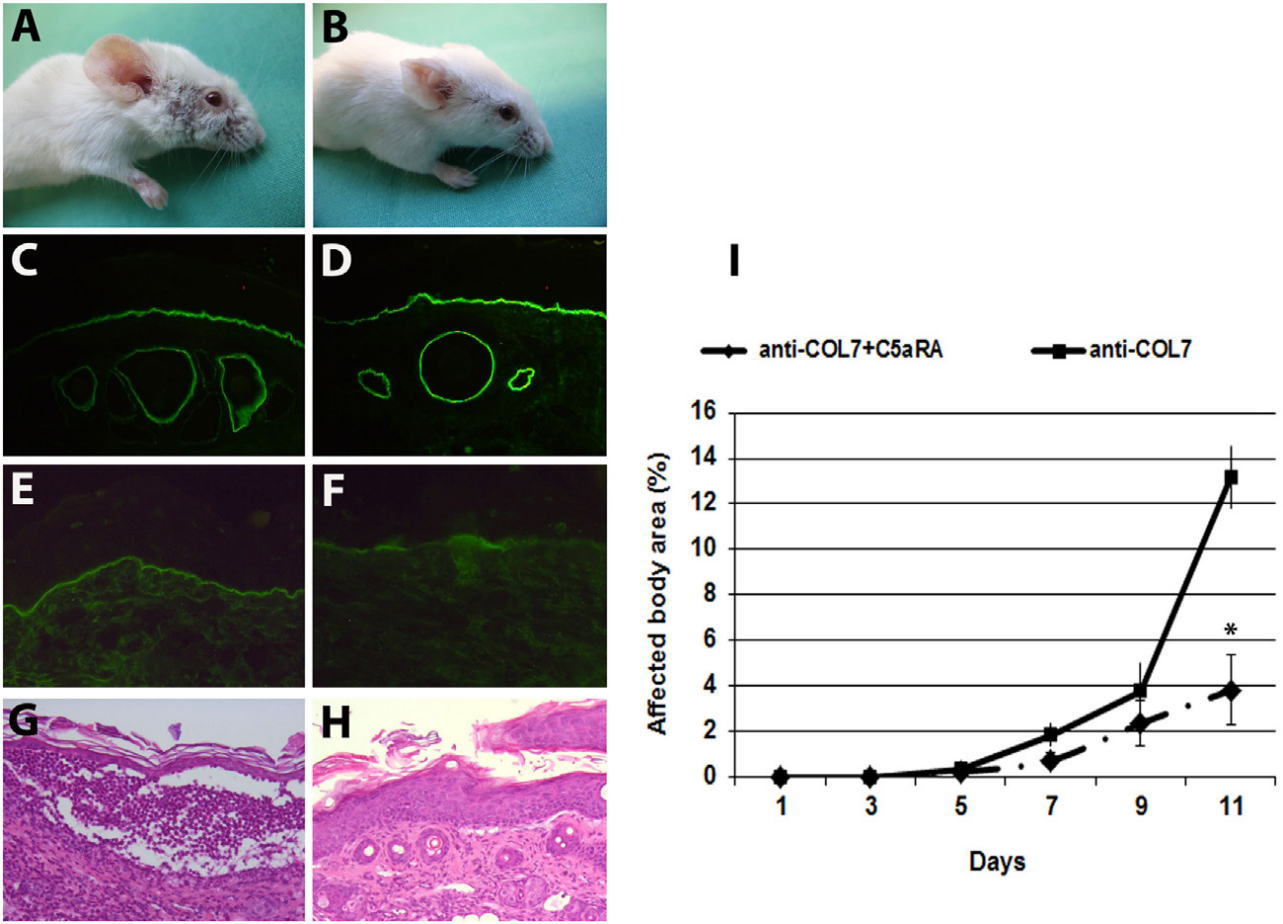
Mice pretreated with a C5a receptor (C5aR) antagonist develop less severe blistering. Skin lesions, including blisters and erosions covered by crusts on the ear, snout, front leg, and around eyes developed in a control mouse injected with pathogenic IgG **(A)**. A BALB/c mouse pretreated with a C5aR antagonist and subsequently challenged with the same dose of antibodies to type VII collagen (COL7) showed less extensive skin lesions **(B)**. Immunofluorescenc analysis of perilesional skin revealed deposition of rabbit IgG at the dermal–epidermal junction of both mice groups **(C,D)**. Deposits of mouse C5 along the dermal–epidermal junction were weak in the skin of the pretreated **(F)** vs non-treated mouse **(E)**. Subepidermal cleavage and neutrophil infiltration was extensive in the skin of control mice **(G)** and less present in the skin of treated mice **(H)**, as revealed by histopathology. Affected body area of C5aRA treated vs control mice is represented as mean ± SEM. *p < 0.05 represents significant difference of disease activity between the two groups **(I)**. These data were obtained from a single experiment (*n* = 4/group).

As the experiments using C5aR1-deficient mice demonstrated that C5a–C5aR1 interactions are critical for blister formation in this model, we pharmacologically targeted the C5aR1 signaling using a C5aRA (27). BALB/c mice were injected with anti-COL7 IgG for EBA induction. One group of mice was pretreated with the C5aRA and showed a less severe blistering phenotype compared to control mice (Figure 5). These findings identify C5aR1 as a potential drug candidate for EBA treatment.

## Discussion

While the importance of C5 and mechanisms leading to C5 cleavage in driving blistering and inflammation in PD are well established, relatively little was known regarding which complement components downstream of C5 cleavage are involved in this process. We here close this knowledge gap, confirming a major role of the C5a/C5aR1 axis and excluding a significant contribution of the MAC in the effector phase of EBA. The insights into the contribution of complement components in EBA were then translated into assessment of treatment in pre-clinical disease models. Specifically, antibodies against fB or C5 prevented onset of blistering and ameliorated disease progression in already established EBA.

Our data also add to the understanding of the complex contribution of the complement system in the pathogenesis of blistering and inflammation in EBA. First, we highlight the predominant role of the C5a/C5aR1 axis, by demonstrating a pronounced amelioration of the blistering phenotype in antibody transfer-induced EBA in mice lacking the C5aR1. These insights into C5a/C5aR1 pathophysiology also shape out current understanding of EBA pathogenesis: the binding of the autoantibodies to COL7 initiates blistering in EBA; one of the immediate subsequent events is the activation of complement, which in antibody transfer-induced EBA occurs within 24 h after anti-COL7 IgG injection (30). Complement activation thus precedes the onset of blistering. Taking into account the effects of C5a on myeloid cells, i.e., induction of migration and activation (31), it is likely that C5a facilitates myeloid cell extravasation into the skin. Furthermore, “C5a can act as a general regulator of Fcgamma receptor (FcγR) expression and the C5aR1 signaling cascade seems to be important for this regulation, suggesting a hierarchical relationship between the two receptors: C5a attracts FcγR-bearing leucocytes, and C5aR transcriptionally regulates expression of inhibitory and activating FcγRs on macrophages to lower the threshold of immune complex activation. Recent studies also suggest the importance of direct cross-talk between C5aR1 and human FcγRs for progression of inflammatory disease, thus highlighting new aspects of this complex interaction” [reviewed in Ref. (32)]. Moreover, the recently discovered negative feedback loop in the C5aR-FcγR cross-talk *via* dectin-1 (18) and galactosylated IgG1 Abs (33) may have an impact on the development of glycoengineered intravenous immunoglobulins (IVIG) analogous to the IVIG preparations administered in autoimmune diseases as an anti-inflammatory therapy (34). However, complement activation significantly, but not exclusively contributes to the formation of this pro-inflammatory milieu and other pro-inflammatory mediators, such as GM-CSF (35) or IL-1β (36), could compensate for the lack of complement-mediated myeloid cell recruitment into the skin. This assumption is further supported by the observation of the occurrence of blistering in the absence of complement activation in (albeit few) outbred mice after immunization with COL7 for EBA induction (37).

Second, our data exclude a significant contribution of the MAC in the induction of inflammation and blistering in EBA. Previously, deposits of human IgG, murine C3, and the MAC were detected in the skin of mice injected with IgG antibodies purified from a patient with severe EBA (38). Furthermore, IgG antibodies from BP patients were able to fix all terminal complement components (C5–C9) resulting in MAC assembly *in vitro* (39). Hence, a contribution of the MAC to pathogenesis of blistering in PD may well have been assumed. Noteworthy, C6-deficient mice are unable to generate functional MAC but can still form C5a. Therefore, our data do not exclude the possibility that MAC might contribute to the skin damage, but support the critical role of the C5a/C5aR1 axis, suggesting that skin inflammation due to leukocyte recruitment and activation by C5a is sufficient to induce blistering.

Since a number of complement-targeting compounds are either in clinical trials or already licensed, our findings can be translated into clinical practice in a relatively timely fashion. More specifically, the humanized anti-C5 antibody (eculizumab) is widely used to treat atypical hemolytic uremic syndrome and paroxysmal nocturnal hemoglobinuria (22). In addition, eculizumab was shown to be safe and well-tolerated in a phase 1 trial in patients with systemic lupus erythematosus (40). These encouraging data suggest that anti-C5 therapy, as well as inhibition of the C5a–C5aR1-axis, could prevent complement-mediated injury in human EBA, and attenuate skin inflammation due to decreased leukocyte recruitment and activation by C5a. In support of this view, the C5aR1 antagonist CCX168 (ChemoCentryx) showed a significant therapeutic effect in a phase 2 study of anti-neutrophil cytoplasmatic antibody-associated renal vasculitis (34). Regarding inhibition of fB, a fragment of the anti-fB antibody 1379 (TA106) is currently available at Taligen Therapeutics/Alexion Pharmaceuticals, but no development intentions have been declared yet (34). Another inhibitor of the alternative pathway, an mAb against factor D, lampalizumab (Roche), has been very recently shown to reduce geographic atrophy progression secondary to age-related macular degeneration (41). Yet, based on our findings of a superior response to preventive vs therapeutic fB inhibition, we envision that complement-targeting treatments may be best employed to maintain the therapeutic response after achieving clinical remission. Furthermore, an alternative mode of drug application involving complement-targeting topical therapy could be potentially very appealing to prevent disease progression in PD. Hence, daily topical application of a purinergic P2X receptor antagonist inhibited complement deposition and choroidal neovascularization in experimental age-related macular degeneration (42).

A serious drawback of (prolonged) C5-directed treatment is the high risk of infections. Deficiency of the terminal complement proteins (C5–C9) is associated with meningococcal infections, and all patients treated with eculizumab are vaccinated against *Neisseria meningitidis*. The selective blockade of either fB or the C5a/C5aR1-axis, which may even be tailored to each individual patient’s needs, is likely to have less side effects than blockade of C5. In addition, especially in BP, where activation of C5 predominantly depends on the classical pathway (13), selective blockade of the classical pathway through C1s inhibition (43), might prove useful for PD treatment.

Different models of EBA are available to mimic the clinical situation in patients, explore disease progression and develop new therapeutic approaches. The use of the antibody transfer model closely resembles the clinical situation and enables the examination of the effector phase of EBA. Testing therapeutic drugs or investigating the pathogenesis of EBA is easily performed because the clinical symptoms are visible within days after the IgG transfer (24). However, antibody transfer models are not suitable for the evaluation of long-term therapeutic effects (24). Recent data brought evidence for a xenogeneic immune reaction to rabbit anti-mouse COL7 IgG, a confounding effect that may contribute to immune complex-driven inflammation and tissue damage in this model, especially at later time points. Therefore, evaluation of results within the first 2 weeks after pathogenic antibody injection is recommended (44). Complex immunization-induced models are more suitable for the investigation of all aspects of autoimmune blistering diseases, including long-term therapeutic intervention (24). In the immunization-induced model of EBA, injection of mice with the immunodominant NC1 domain of COL7 leads to anti-COL7 autoantibody-production in most mice strains, whereas development of subepidermal blistering is restricted to certain strains. Furthermore, comparison of the autoantibody response in EBA-susceptible and -resistant mice showed an association of clinical disease with formation of complement-fixing (IgG2) anti-COL7 antibodies (8). Hence, we emphasize that complement-blocking therapy may ameliorate disease progression in the EBA immunization-induced model. However, due to the dependency on complement-fixing antibodies, activation by the classical pathway might be more relevant in the immunization-model. Thus, selective blockade of the classical pathway might prove more useful than fB inhibition. Similar to the antibody transfer model, anti-C5 therapy, as well as inhibition of the C5a–C5aR1-axis, would probably efficiently ameliorate complement-mediated injury in immunization-induced EBA and attenuate skin inflammation due to decreased leukocyte recruitment and activation by C5a. However, overwhelming the natural tolerance against skin proteins remains challenging in immunization models. In addition, the effects of adjuvants during immunization remain largely unknown (24).

In conclusion, our study identified promising candidate molecules for complement-directed therapeutic concepts in EBA. Novel therapeutic agents are required in autoimmune disease, as treatment strategies of most antibody-mediated diseases, including EBA, are unspecific and resume to prolonged administration of systemic corticosteroids and additional immunosuppressants, being frequently associated with severe side effects, including death (45). At this point, the clinically available anti-C5 antibody eculizumab is the most promising candidate for clinical trials in EBA.

## Ethics Statement

This study was carried out in accordance with the recommendations of European Community rules for animal care. The protocol was approved by the Ministry for Energy, Agriculture, the Environment and Rural Areas, Kiel, Schleswig-Holstein and the Government of Middle Franconia, Germany.

## Author Contributions

SM, DZ, and FN conceived and designed the research studies. SM and MH performed experiments, collected, assembled, and interpreted data. YW, JMT, VMH, BPM, JK, and RJL provided reagents, performed data analysis and interpretation. SM, RJL, and FN wrote the manuscript. All authors contributed to editing, reviewed and approved the final manuscript.

## Conflict of Interest Statement

The authors declare a potential conflict of interest and state it below. RJL has received funding from True North Therapeutics, a manufacturer of the C1s antibody TNT003. VMH and JMT receive royalties from Alexion Therapeutics.

## Abbreviations

COL7, type VII collagen; EBA, epidermolysis bullosa acquisita; C5aR, C5a receptor; MAC, membrane attack complex; fB, factor B; PD, pemphigoid diseases; IF, immunofluorescence; FcγR, Fc gamma receptor.
